# Survival Outcomes After Radiotherapy for the Treatment of Synchronous Oligometastatic Prostate Cancer

**DOI:** 10.1001/jamanetworkopen.2022.35345

**Published:** 2022-10-07

**Authors:** Linda My Huynh, Benjamin T. Bonebrake, Charles Enke, Michael J. Baine

**Affiliations:** 1 Department of Radiation Oncology, Fred & Pamela Buffett Cancer Center, University of Nebraska Medical Center, Omaha; 2 School of Medicine, University of Nebraska Medical Center, Omaha

## Abstract

This cohort study assesses biochemical progression-free survival among patients receiving radiotherapy for the treatment of synchronous oligometastatic prostate cancer.

## Introduction

With improvement in imaging for prostate cancer, diagnosis of oligometastatic disease has substantially increased,^1^ and questions concerning metastasis-directed therapy for biochemical progression-free survival (bPFS) and overall survival have surfaced. Evidence suggests ablative radiotherapy may delay disease progression and defer systemic therapies.^2^ However, previous explorations have focused on metachronous metastases, with no substantial data on synchronous metastases.^3^ The present cohort study sought to assess bPFS among patients receiving radiotherapy for synchronous oligometastatic prostate cancer.

## Methods

From May 1, 2011, to May 30, 2021, 43 patients received radiotherapy for synchronous oligometastatic prostate cancer (defined as ≤5 metastatic sites). Demographic and oncological characteristics, treatment, adverse events (AEs), and bPFS were retrospectively assessed under approved University of Nebraska Medica Center Institutional Review Board protocol, and a waiver of consent was granted because the study used deidentified data. Adverse events were recorded using Common Terminology Criteria for Adverse Events (CTCAE) grades 1 to 5. All patients received definitive doses of radiotherapy to the prostate and metastatic sites with stereotactic body radiotherapy, conventional fractionation, and/or integration of simultaneous integrated boosts in a single treatment course. All patients had metastatic castration-sensitive prostate cancer and received a finite course of androgen deprivation therapy (ADT). Patients treated before 2017 received continuous ADT; those treated from 2018 to 2021 received novel hormonal therapies as standard of care. Patients were subcategorized into pelvic lymph node metastases only (N1-only) or para-aortic nodal and/or bone metastases (M1) groups.

The primary outcome was bPFS, defined as any prostate-specific antigen level increase of 2 ng/mL or greater over nadir and assessed via Kaplan-Meier and Mantel-Cox analyses. Patients were censored at the date of last follow-up. All hypothesis tests were 2-sided, with *P* < .05 considered statistically significant. All statistical analysis was conducted using IBM SPSS Statistics software, version 25 (IBM Corp).

## Results

Among 43 participants, the mean (SD) age was 66.7 (8.6) years; 37 (86.0%) were White; the pretreatment prostate-specific antigen level was 53.3 (75.9) ng/mL. The mean (SD) duration of ADT was 2.3 (0.5) years, and all participants completed ADT by the time of analysis. Grade 3 and 4 CTCAEs were reported in 1 patient (2.3%) per group; no grade 5 CTCAEs were reported.

Of 43 patients, 10 (23.3%) had M1 disease, and 33 (76.7%) had N1-only disease. Of those with M1 disease, 2 patients (20.0%) had M1a (metastases to lymph nodes away from groin area) and 8 (80.0%) had M1b (bone metastases). Patients with M1 disease were significantly older than those with N1-only disease (Table). There were no significant differences in pretreatment prostate-specific antigen levels, Gleason scores, or rates of bPFS. At median follow-up of 4 years (range, 2-12 years), 2 of 10 patients (20.0%) with M1 disease and 5 of 33 patients (15.2%) with N1-only disease experienced biochemical failure. All known failure locations were local. The rate of bPFS was not significantly different (80% for M1 vs 85% for N1-only; χ^2^ = 1.047; Mantel-Cox *P* = .55) (Figure). The mean (SE) Kaplan-Meier estimate for survival time was 10.60 (0.81) years. Overall mortality was significantly higher in the M1 group vs the N1-only group (3 patients [30.0%]) vs 0 patients; *P* = .01); 1 patient died of unrelated causes. The mean (SD) time to death was 5.8 (1.2) years.

**Table.** Participant Clinicodemographic Characteristics

Characteristic	Patients, No./total No. (%)		*P* value
	M1 group (n = 10)	N1-only group (n = 33)	
Age, mean (SD), y	72.6 (6.5)	66.3 (7.8)	.01
Race and ethnicity			
Asian	0	2/33 (6.1)	.39
Black	0	3/33 (9.1)	
Hispanic	0	0	
White	10/10 (100)	27/33 (81.8)	
Other	0	1/33 (3.0)	
Pretreatment PSA level, mean (SD), ng/mL	19.8 (7.2)	66.0 (89.7)	.23
Time to last follow-up, mean (SD), y	3.7 (2.5)	5.0 (3.3)	.53
BMI, mean (SD)	34.1 (4.4)	30.2 (5.3)	.14
No. of metastatic lesions, mean (SD)	1.8 (1.3)	2.0 (1.3)	.67
Duration of ADT, mean (SD), y	2.5 (0.7)	2.2 (0.4)	.30
Family history of prostate cancer	2/10 (20.0)	5/33 (15.2)	.90
Gleason summary score			
7	0	6/33 (18.2)	.40
8	2/9 (22.2)	7/33 (21.2)	
9	6/9 (66.7)	17/33 (51.5)	
10	1/9 (11.1)	3/33 (9.1)	
Postradiotherapy biochemical failure	2/10 (20.0)	5/33 (15.2)	.79
Postradiotherapy biochemical failure or salvage treatment	3/10 (30.0)	12/33 (36.4)	.80
Died	3/10 (30.0)	0	.01
Unavailable for follow-up	1/10 (10.0)	4/33 (12.1)	.59

Abbreviations: ADT, androgen deprivation therapy; BMI, body mass index (calculated as weight in kilograms divided by height in meters squared); M1, para-aortic nodal and/or bone metastases; N1-only, pelvic lymph node metastases only; PSA, prostate-specific antigen.

SI conversion factor: To convert PSA from nanograms per milliliter to micrograms per liter, multiply by 1.0.

**Figure.**
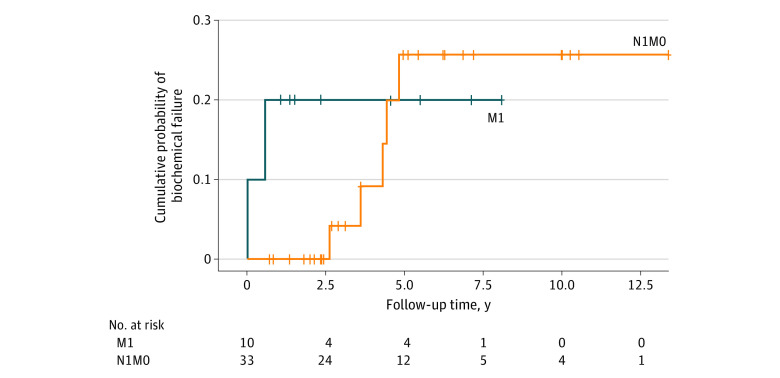
Kaplan-Meier Analysis of Biochemical Progression-Free Survival Among Patients in M1 and N1-Only Groups Tick marks represent times of censoring. M1 indicates para-aortic nodal and/or bone metastases (M1 group); and N1M0, pelvic lymph node metastases only (N1-only group).

## Discussion

Distinct from the STOMP,^4^ ORIOLE,^5^ and SABR-COMET^6^ clinical trials, the findings of this cohort study represent great potential for metastasis-directed therapy. While previous studies either treated only the prostate in patients with synchronous metastases or treated metachronous metastases after definitive treatment, the present results support simultaneous treatment of synchronous metastases. Although limited by its single-center, retrospective design, this study’s findings promote the use of radiotherapy for synchronous metastases, with bPFS rates of approximately 80%, which is consistent with several studies of metastasis-directed therapy.^2,3,4^ Furthermore, durability of response, morbidity, and control rates did not significantly differ between N1-only and M1 groups. Overall, radiotherapy was well tolerated, with grade 3 to 5 CTCAEs reported in fewer than 5% of patients. Although testosterone recovery was undocumented, ADT use was finite, with a mean duration of 2.3 years.

Several prospective studies on metastasis-directed therapy for the treatment of oligometastatic M1a prostate cancer are ongoing.^6^ To ascertain the best treatment approach for these patients, high-quality phase 3 clinical trials are still needed.
